# Glucocorticoids combined with rituximab treatment for IgG4-related tubulointerstitial nephritis with diabetic nephropathy: a case report

**DOI:** 10.1515/med-2026-1528

**Published:** 2026-09-21

**Authors:** Tianshu Wang, Jingjing Zhang, Yaguang Xu

**Affiliations:** Department of Nephrology, The Second People’s Hospital of Liaocheng, Linqing City, Shandong Province, China

**Keywords:** IgG4-related disease, IgG4-related kidney disease, IgG4-related tubulointerstitial nephritis, rituximab, acute kidney injury

## Abstract

**Objectives:**

IgG4-related disease is a chronic immune-mediated disorder featuring elevated serum IgG4 levels and the tissue infiltration of IgG4-positive plasma cells. Kidney involvement in IgG4-related disease, known as IgG4-related kidney disease, most commonly manifests as tubulointerstitial nephritis.

**Case Presentation:**

A 55-year-old male presented with acute kidney injury (creatinine=816 μmol/L), severe anemia, lymphadenopathy, and high IgG4 levels (13.89 g/L). Renal MRI and Chest CT showed diffuse renal abnormalities and mediastinal lymphadenopathy. Renal biopsy confirmed IgG4-related tubulointerstitial nephritis, complicated by IgA nephropathy and diabetic nephropathy. Initial glucocorticoid treatment was prescribed followed by 1.8 g rituximab consolidation therapy. Over 32 months follow-up, the patient’s renal function significantly improved (creatinine 215 μmol/L) and his serum IgG4 levels normalized (0.89 g/L).

**Conclusions:**

This success of this case suggests that combination therapy may be a reasonable option in selected cases, and underscores the importance of an early diagnosis, pathological confirmation, and standardized immunosuppressive therapy for improving the outcomes in IgG4-related kidney disease.

## Introduction

Immunoglobulin G4-related disease (IgG4-RD) is a chronic inflammatory disorder caused by the abnormal functioning of the immune system, and is characterized by markedly elevated serum IgG4 levels and a dense infiltration of IgG4-positive plasma cells across multiple organs [1], 2]. This disease can affect various organs, including, but not limited to, the kidneys, liver, gallbladder, pancreas, lungs, bile ducts, and thyroid gland. When the kidneys are involved, the condition is known as IgG4-related kidney disease (IgG4-RKD). The typical pathology of IgG4-RKD features tubulointerstitial nephritis. Clinically, it may present as acute or chronic kidney injury, a mass-like kidney lesion, or both, leading to renal impairment and symptoms such as anemia, low-grade fever, and fatigue [3], 4]. Without prompt diagnosis, the disease may ultimately advance to chronic renal failure or uremia, with the patient then requiring dialysis. Therefore, an early diagnosis and treatment are crucial to slow the disease progression and protect kidney function [5]. In this study, the clinical data of a patient with IgG4-related tubulointerstitial nephritis (IgG4-TIN) complicated by IgA nephropathy and diabetic nephropathy (DN) were reviewed, and are summarized and discussed in this case report. This case report was approved by the Ethics Committee of the Second People’s Hospital of Liaocheng (2025-LG-077). Patient consent was obtained for the use of personal information.

## Case presentation

This case report concerns a 55-year-old male patient, who was admitted to a local community hospital on June 8, 2022 with complaints of “fatigue and blurred vision in both eyes”. Laboratory tests performed during the patient’s hospitalization period revealed he had anemia (hemoglobin=71 g/L) and thrombocytopenia (platelets=90 × 10^9^/L). Following the administration of symptomatic treatment, which included glycerol, fructose, and iron supplementation, the patient exhibited a slight improvement in symptoms and was discharged on June 15, 2022. However, the following day (June 16), the patient presented at our hospital’s Oncology Outpatient Clinic. Subsequently, evaluation tests were performed, including blood tests, and revealed the patient had a hemoglobin level of 61 g/dL, platelet count of 79 × 10^9^/L, urinary protein level of 409 mg/L, prothrombin time of 14.1 s, and D-dimer level of 0.87 mg/L. Iron metabolism tests were also performed and revealed the patient had ferritin levels of 293.4 ng/mL, vitamin B12 levels of 470 pg/mL, and folate levels of 8.24 ng/mL. Urinalysis showed occult blood 1+, glucose 3+, and negative protein. Bone marrow aspiration was subsequently recommended; however, the patient declined and left the hospital.

On the next day (June 17, 2022), the patient presented to yet another hospital, this time with epistaxis. Laboratory tests were performed, indicating that he had an erythrocyte sedimentation rate (ESR) of 150 mm/h, and the following blood biochemistry results: total protein: 89.5 g/L; albumin: 40.9 g/L; globulin: 48.6 g/L; creatinine: 816 μmol/L; blood urea nitrogen (BUN): 36.5 mmol/L; serum potassium: 5.78 mmol/L. Nasal packing was employed to achieve hemostasis, and it was recommended that the patient should be hospitalized for hemodialysis; however, the patient declined this course of action. On June 18, 2022, the patient was finally admitted to our hospital, having experienced ten days of fatigue and one day of detected renal dysfunction.

The patient’s medical history was taken, revealing he had experienced a weight loss of approximately 10 kg over the past six months, and had a history of type 2 diabetes mellitus for 6 years and hypertension for 3 years, which were generally well-controlled with oral medications. Also, the previous month in May 2022 he had presented to other hospital with blurred vision in the left eye, and was diagnosed with bilateral retinal degeneration. Upon admission to our hospital, a range of further tests and examinations were carried out on the patient to assess his condition, with the results reported below.

Physical examination: body temperature: 37.2 °C; heart rate: 94 bpm; respiration: 19 bpm; blood pressure: 162/99 mmHg; anemic appearance; multiple lymph nodes palpable in the neck and axillae, firm in consistency, mobile, and non-tender; pale conjunctiva; and the presence of gauze packing in the left nasal cavity with minimal bloody discharge visible.

Laboratory tests: Complete blood count: White blood cells: 3.53 × 10^9^/L, red blood cells: 1.59 × 10^12^/L, hemoglobin: 50 g/L, platelet count: 105 × 10^9^/L, C-reactive protein: 14.2 mg/L. Immunological: Complement C3: 0.245 g/L, complement C4: <0.0167 g/L, immunoglobulin G: 32.5 g/L, immunoglobulin E: 213 IU/mL, antinuclear antibody (ANA): 1:320 (nuclear homogenate pattern), anti-phospholipase a2 receptor antibody (anti-PLA2R): 1.72 RU/mL; anti-neutrophil cytoplasmic antibody, anti-glomerular basement membrane antibody, extractable nuclear antigen antibody, and anti-dsDNA antibody all negative; serum immunofixation electrophoresis showed no abnormalities. Light chain analysis: κ light chain: 10.26 g/L, λ light chain: 5.74 g/L, κ/λ ratio within normal range. Coagulation function: fibrinogen: 4.25 g/L, D-dimer: 0.96 mg/L. Renal function and electrolytes: creatinine: 817 μmol/L, BUN: 32.63 mmol/L; carbon dioxide: 17.20 mmol/L, potassium: 4.92 mmol/L, calcium: 2.00 mmol/L, phosphorus: 1.86 mmol/L. Urinalysis: urinary creatinine: 4.1 mmol/L, urinary microalbumin/creatinine ratio: 1.97 mg/mmol, urinary immunoglobulin G: 19.25 mg/L; Urinalysis: occult blood (±), urinary glucose (++), urinary protein negative, pH 7; urinary Bender’s protein negative. Other: parathyroid hormone: 176.80 pg/mL; Normal thyroid function; Negative for infectious disease screening.

**Figure 1:**
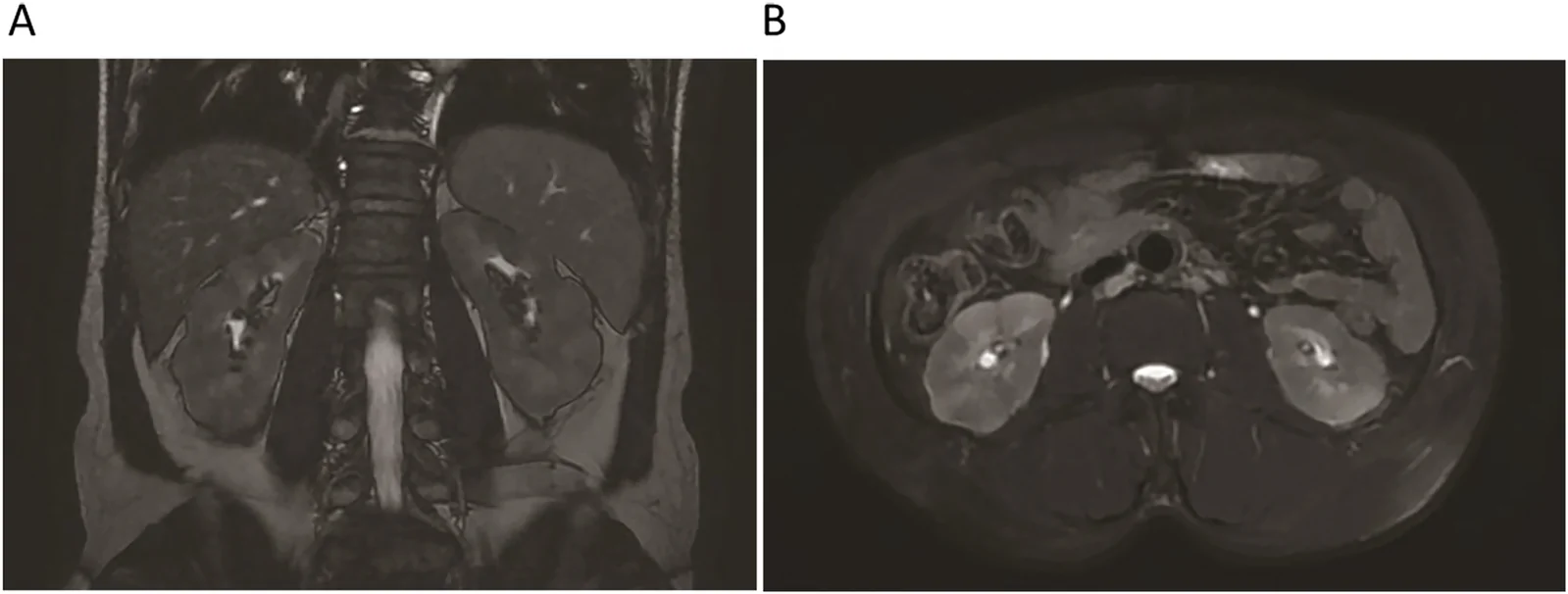
Magnetic resonance imaging of the kidneys; the presence of diffuse signal abnormalities in both kidneys may indicate potential nephritis or nephrotic changes.

Imaging findings: Chest CT: Enlarged mediastinal and bilateral hilar lymph nodes; Ground-glass nodule in the left upper lobe; multiple solid nodules in both lungs. Abdominal and urological ultrasound: intrahepatic calcifications; heterogeneous renal parenchymal echogenicity in both kidneys. Cardiac echocardiography: enlarged left atrium, thickened left ventricular wall, mild aortic and tricuspid valve regurgitation. Renal MRI: diffuse signal abnormalities in both kidneys, suggestive of nephritis or nephropathy (Figure 1). ECT bone scan: increased metabolic activity in bilateral knees and the right ankle, considered to be the result of degenerative changes.

**Figure 2:**
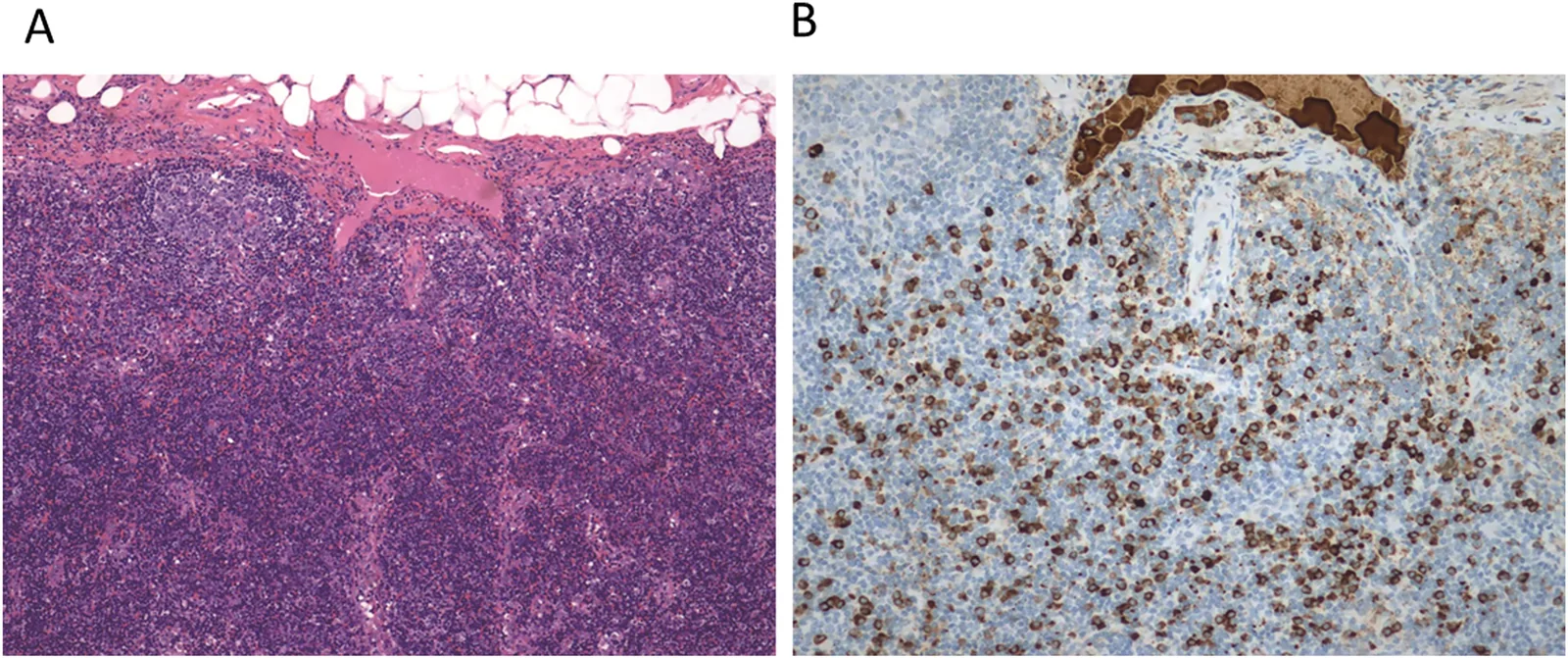
Pathological findings of the left submandibular lymph node, revealing lymph node reactive hyperplasia with IgG4-positive plasma cell proliferation. A: Tissue at medium magnification; B: IgG4 immunohistochemistry.

Following these tests, the patient received a blood transfusion, followed by treatment for anemia, management of blood pressure, and treatment for acidosis. Bone marrow aspiration revealed the patient had thrombocytopenia. His serum IgG4 level was found to be 13.89 g/L (reference range: 0.039–0.86 g/L). Subsequent ultrasound of the neck revealed the presence of bilateral parotid gland nodules and diffuse lesions of the submandibular gland, for which a left submandibular lymph node excision procedure was performed on June 27, 2022. A comprehensive pathology evaluation revealed reactive lymphoid hyperplasia, accompanied by IgG4-positive plasma cell proliferation (Figure 2).

**Figure 3:**
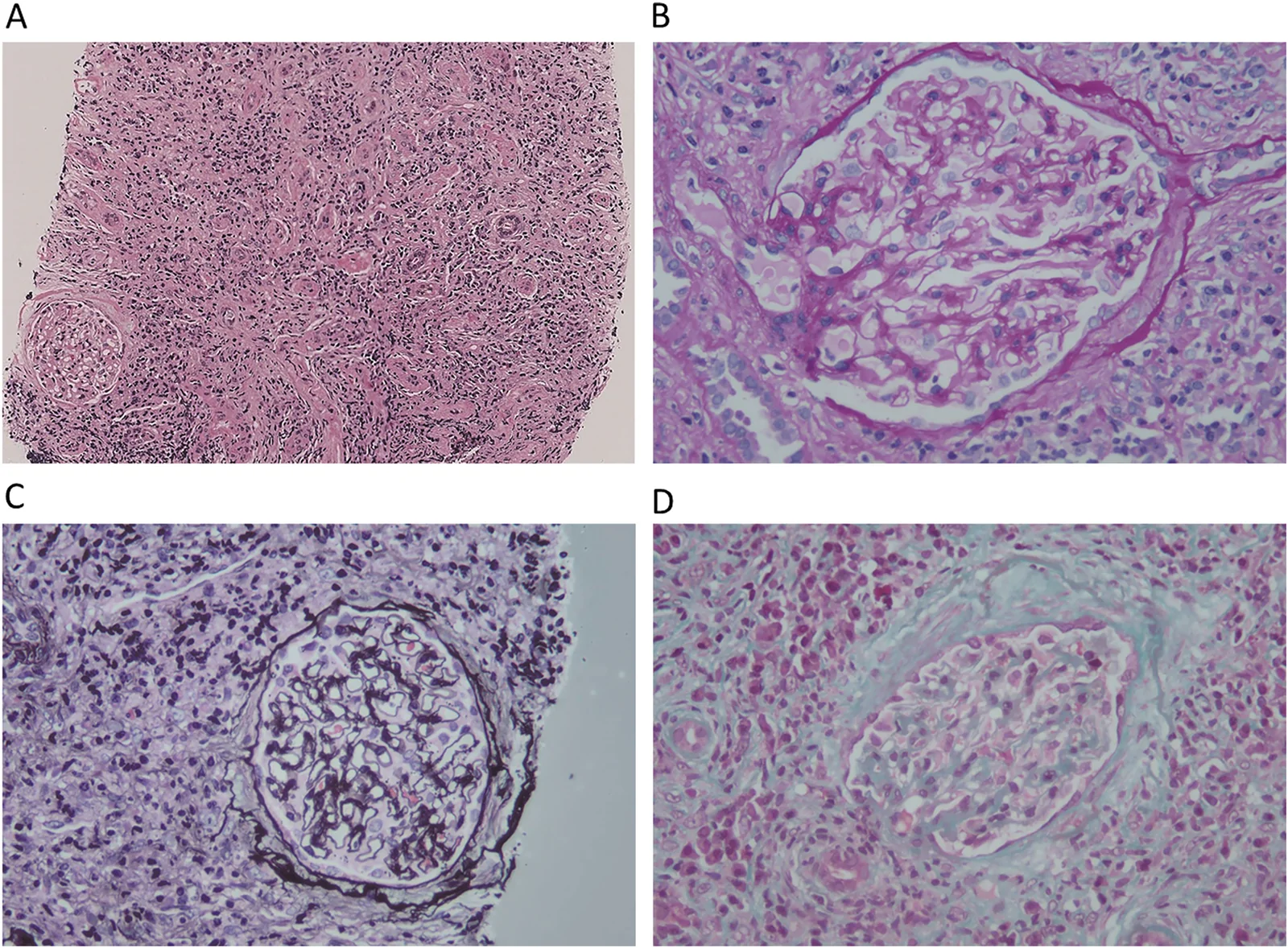
Renal biopsy light microscopy results: (A) Hematoxylin and eosin staining ( × 100) showing diffuse interstitial infiltration by plasma cells; (B) periodic acid schiff staining ( × 400) showing tubular atrophy and interstitial fibrosis; (C) periodic acid-silver methenamine staining ( × 400) showing no evidence of glomerular basement membrane duplication; (D) Masson’s trichrome staining ( × 400) showing interstitial fibrosis with a lamellar pattern.

The patient underwent renal histopathology on July 6, 2022, which revealed a complex renal lesion. Light microscopy revealed mild mesangial cell proliferation and matrix expansion in the glomeruli, accompanied by hemosiderin deposition in the mesangial area. The patient’s tubular epithelial cells exhibited granular degeneration with diffuse tubular atrophy. The renal interstitium exhibited substantial inflammatory cell infiltration, predominantly plasma cells, accompanied by storiform fibrosis with a reticular pattern (Figure 3).

**Figure 4:**
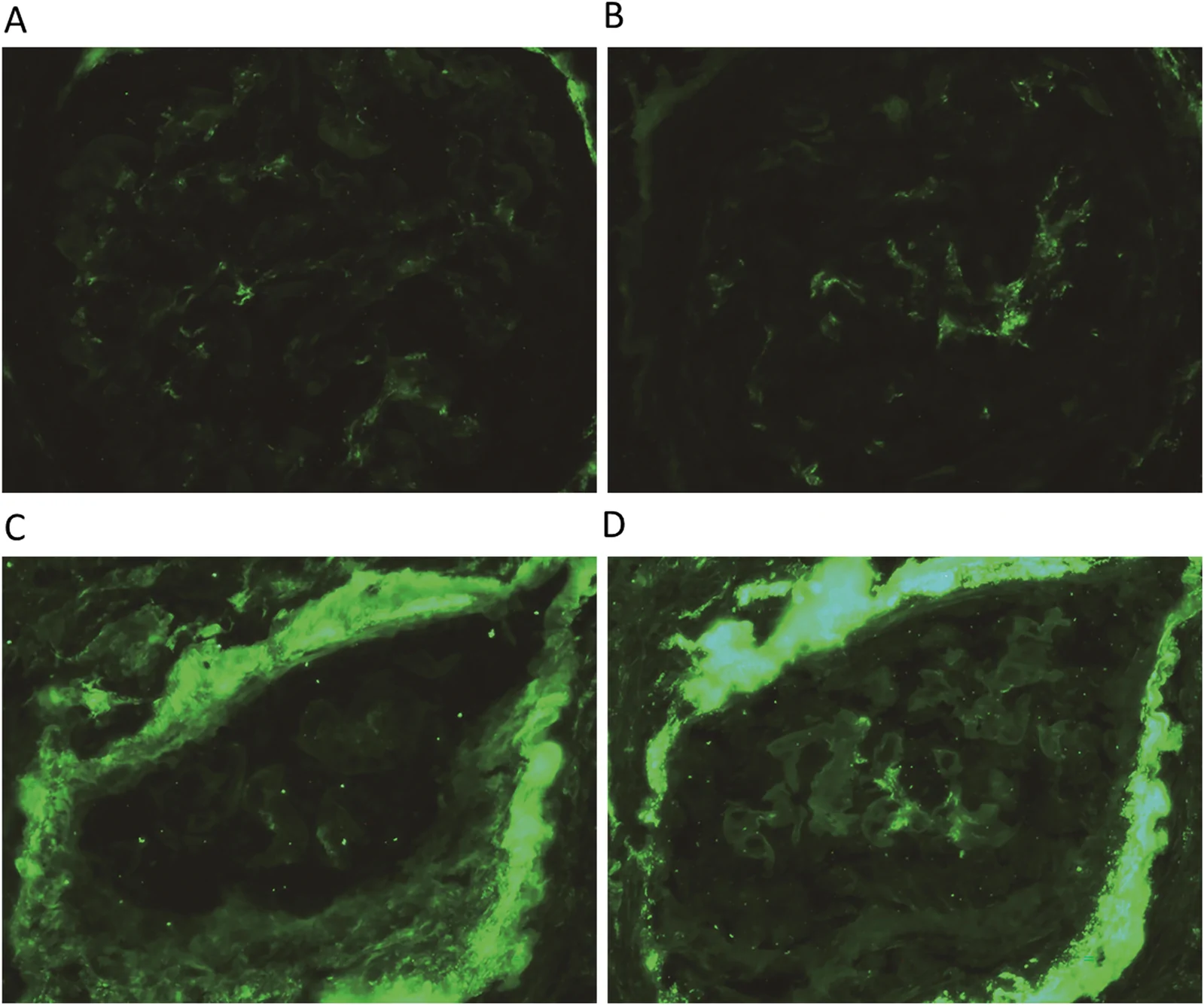
Renal biopsy immunofluorescence: (A) IgA (+); (B) C3 (+); (C) kappa (+/−); (D) lambda (+/−).

Immunofluorescence detection was positive for IgA and C3, with weak positivity for κ and λ light chains (Figure 4).

**Figure 5:**
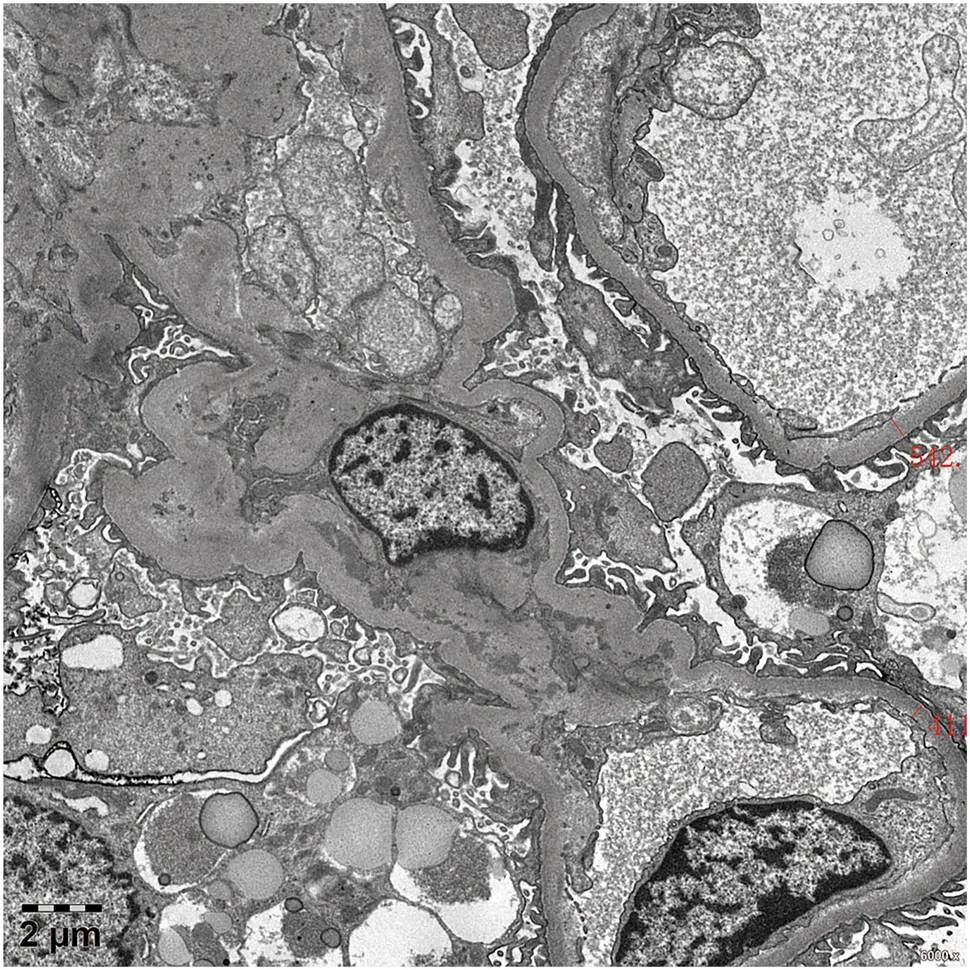
Renal biopsy electron microscopy ( × 6,000): The initial findings revealed a mild homogeneous thickening of the glomerular basement membrane, the segmental fusion of podocytes, and marked fusion in some areas. Additionally, high-density electron-dense deposits were visible in the mesangial areas.

**Figure 6:**
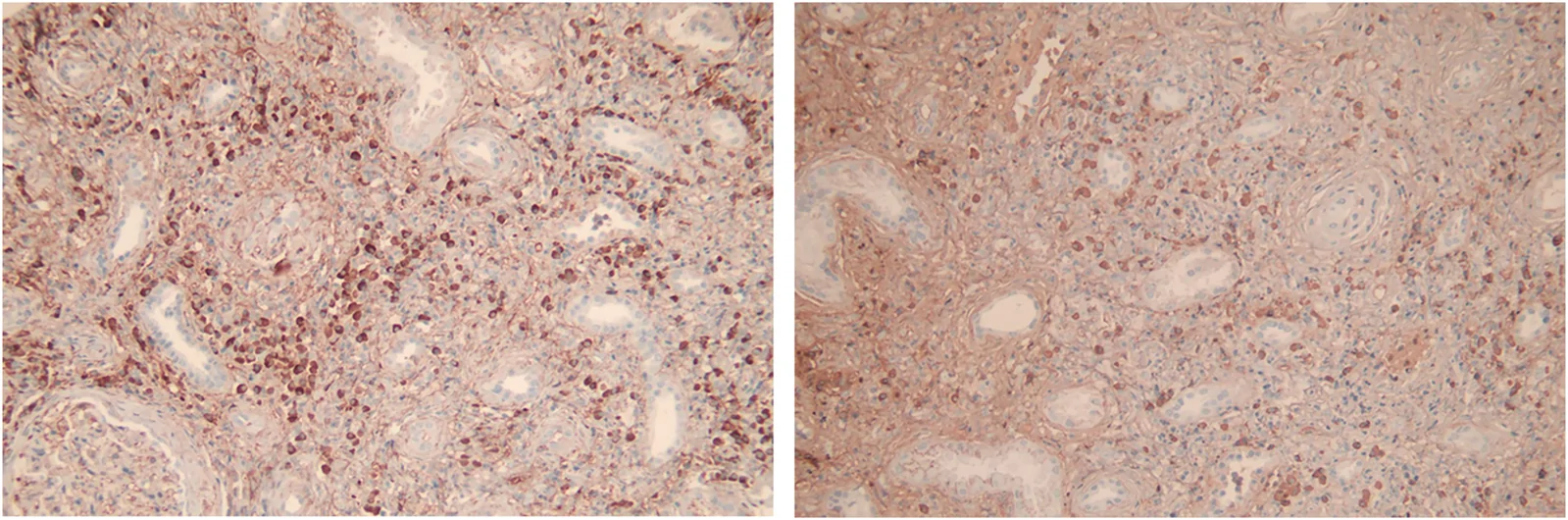
Renal biopsy immunohistochemistry results: (A) IgG, whereby the presence of focal and patchy positive staining in plasma cells within the renal interstitium could be observed. (B) IgG4; whereby the presence of focal positive staining in plasma cells within the renal interstitium could be observed, with a count of >40 positive cells per high-power field. The IgG4/IgG-positive cell ratio was >40 %, indicating a significant proportion of IgG4-positive cells in the sample.

Electron microscopy analysis revealed a mild homogeneous thickening of the glomerular basement membrane, the segmental fusion of podocytes, and a deposition of electron-dense material in the mesangial area (Figure 5). Immunohistochemistry tests further demonstrated a multifocal infiltration of CD38-, IgG-, and IgG4-positive plasma cells in the renal interstitium, with >40 IgG4-positive cells per high-power field and an IgG4/IgG-positive cell ratio of >40 % (Figure 6).

In light of the aforementioned pathological findings, the following diagnoses were confirmed: 1. IgG4-TIN, which was considered the primary cause of acute kidney injury (AKI). This diagnosis was supported by: (a) the presence of significant plasma cell infiltration, i.e., >40 IgG4+ cells/HPF and an IgG4/IgG ratio>40 %; (b) the elevated serum IgG4 level (13.89 g/L); and (c) as the patient showed a rapid improvement in renal function after glucocorticoid therapy (creatinine decreased from 817 to 522 μmol/L within one week). 2. IgA nephropathy. This diagnosis was based on the immunofluorescence imaging showing dominant IgA and C3 deposition in the mesangium; however, its contribution to renal dysfunction was likely minor given the absence of significant hematuria or proteinuria. 3. Diabetic nephropathy (DN). This diagnosis was supported by a consideration of the patient’s long-standing type 2 diabetes (6 years), the observation of glomerular basement membrane thickening upon electron microscopy imaging, and his clinical history.

On July 15, 2022, the patient was started on a course of immunosuppressive therapy, specifically prednisone acetate at 40 mg once daily. Concurrently, the patient’s insulin regimen was adjusted with the objective of achieving optimal glycemic control. Despite the patient’s markedly elevated serum creatinine level, they remained hemodynamically stable with a normal urine output, no signs of heart failure, no gastrointestinal symptoms, and no life-threatening electrolyte disturbances. Consequently, emergency hemodialysis was not initiated during their hospitalization. Furthermore, within one week, the patient’s serum creatinine level had decreased to 522 μmol/L, and the patient was subsequently discharged on July 22, 2022. Following discharge, the patient was prescribed regular oral corticosteroids, which were gradually tapered over time. The patient’s current medication regimen consists of a daily dose of 7.5 mg of prednisone.

To further manage the patient’s condition, multiple rituximab treatments were initiated on August 1, 2023, with a cumulative dosage of 1.8 g. The specific dates and doses of rituximab administration were as follows: 0.5 g on August 5, 2023; 0.3 g on August 31, 2023; 0.2 g on October 5, 2023; 0.2 g on November 8, 2023; 0.3 g on January 13, 2025; 0.3 g on February 25, 2026. At the most recent follow-up on March 28, 2026, the patient’s IgG4 level had decreased to 0.89 g/L, while their serum creatinine remained stable at approximately 215 μmol/L, and the patient’s condition was considered stable (Table 1).

**Table 1:** Treatment timeline and key clinical responses.

Date	Event/intervention	Prednisone dose	Rituximab dose (cumulative)	Serum creatinine, μmol/L	Serum IgG4, g/L	24-h urinary protein, g	Remarks
June 18, 2022	Hospital admission	–	–	817	Not tested	–	AKI, anemia, lymphadenopathy
July 6, 2022	Renal biopsy	–	–	–	–	–	Confirmed IgG4-TIN + IgA nephropathy + diabetic nephropathy
July 15, 2022	Glucocorticoid initiation	40 mg/day	–	790	13.89	0.75	Start of first-line therapy
July 22, 2022	Discharge	40 mg/day (tapering planned)	–	522	–	0.68	Significant improvement within 1 week
August 2022 – July 2023	Gradual steroid tapering	Reduced from 40 to 7.5 mg/day	–	Gradually improved to 285–496	Declining but still elevated	0.40–0.76	Maintenance phase
August 1, 2023	Decision for rituximab consolidation	7.5 mg/day	0 g	306	4.55	0.71	Rituximab as consolidation therapy to reduce relapse risk and enable further steroid tapering
August 5, 2023	Rituximab dose 1	7.5 mg/day	0.5 g (total 0.5 g)	–	–	–	–
August 31, 2023	Rituximab dose 2	7.5 mg/day	0.3 g (total 0.8 g)	289	2.79	0.45	–
October 5, 2023	Rituximab dose 3	7.5 mg/day	0.2 g (total 1.0 g)	296	2.24	0.53	–
November 8, 2023	Rituximab dose 4	7.5 mg/day	0.2 g (total 1.2 g)	273	2.03	0.41	–
January 13, 2025	Rituximab dose 5	7.5 mg/day	0.3 g (total 1.5 g)	235	2.58	0.65	–
February 25, 2026	Rituximab dose 6	7.5 mg/day	0.3 g (total 1.8 g)	229	1.81	0.52	–
March 28, 2026	Last follow-up	5 mg/day	–	215	0.89	0.36	Stable renal function, normalized IgG4

## Discussion

IgG4-RD is a multisystem disorder that frequently involves the kidneys. The renal pathology of this disease manifests in various forms, with IgG4-TIN being the most common [6]. A broad spectrum of renal manifestations may occur, including membranous glomerulonephritis (with or without IgG4-TIN), IgA nephropathy, membranoproliferative glomerulonephritis, IgG4-related plasmacytoma arteritis, and IgG4-related retroperitoneal fibrosis or ureteral pseudotumor [7]. IgG4-RKD mainly affects elderly males. A significant proportion of patients – about 80 % – show symptoms of hypergammaglobulinemia, including elevated serum total IgG and/or IgG4 levels [4], [8], [9], [10]. It is important to note that elevated serum IgG4 is not exclusive to IgG4-RD, as elevated levels are also seen in roughly 5 % of healthy individuals and 10 % of pancreatic cancer patients [11]. Moreover, various other laboratory abnormalities have been identified in IgG4-RD cases, including hypocomplementemia (low C3 and/or C4), increased serum IgE levels, and peripheral blood eosinophilia, with rates ranging from 33 % to 48 %. About 30 % of patients test positive for ANA, usually at low titers [12].

The diagnostic criteria for IgG4-RD are continuously being refined as knowledge and experience of IgG4-RD grow. The Japanese Society of Nephrology’s established criteria emphasize histopathological features as the primary basis or diagnosis, including extensive lymphoplasmacytic infiltration, reticular fibrosis, and occlusive phlebitis. Immunohistochemistry diagnosis requires at least 10 IgG4+ plasma cells per high-power field (HPF), or an IgG4+/IgG+ plasma cell ratio of 40 % or greater [13], 14]. Additional supportive criteria for diagnosing IgG4-RD are elevated serum IgG4 levels (≥135 mg/dL), imaging evidence of renal morphological or density abnormalities, renal impairment, and a similar involvement of other organs [4], 15]. The presence of hypoalbuminemia, hypergammaglobulinemia, elevated IgE, eosinophilia, and a positive ANA test may serve as secondary indicators, although their diagnostic significance require further validation [16]. While the Japanese criteria for IgG4-RD are considered more stringent than other criteria, they can result in its underdiagnosis in some patients. Conversely, the Mayo Clinic criteria do not require histopathological confirmation, thereby improving the diagnostic sensitivity, but potentially increasing the risk of misdiagnosis [17]. In the present case, immunohistochemistry revealed the presence of over 40 IgG4+ plasma cells per HPF in the renal interstitium, with an IgG4+/IgG+ plasma cell ratio exceeding 40 %. This finding aligns with the diagnostic threshold proposed by the Japanese Society of Nephrology (≥10/HPF and ≥40 % ratio), indicating a positive diagnosis. Furthermore, the renal biopsy revealed characteristic storiform fibrosis with a reticular pattern in the interstitium, a key feature of IgG4-RKD. Despite meticulous examination of the renal tissue, occlusive phlebitis was not definitively identified, which is not uncommon in IgG4-TIN, as it is more frequently observed in other organs, such as the pancreas or salivary glands. The mild mesangial IgA and C3 deposition observed upon immunofluorescence supported a diagnosis of concurrent IgA nephropathy, while the homogeneous thickening of the glomerular basement membrane observed upon electron microscopy imaging, together with consideration of the patient’s long-standing diabetes, supported the diagnosis of diabetic nephropathy. These overlapping features were distinguished from IgG4-TIN by their distinct glomerular and basement membrane changes. In contrast, the acute tubulointerstitial injury was predominantly driven by the IgG4-related process.

Given the patient presented with lymphadenopathy, anemia, thrombocytopenia, hypergammaglobulinemia, and AKI with tubulointerstitial nephritis, it was essential to carefully exclude several alternative potential diagnoses. In this regard, lymphoma was initially considered a possible diagnosis due to the presence of mediastinal and peripheral lymphadenopathy, as well as due to the systemic symptoms the patient displayed. However, subsequent evaluation by lymph node biopsy revealed reactive lymphoid hyperplasia without the presence of atypical cells or clonality. Furthermore, bone marrow aspiration tests were performed but did not show any evidence of lymphomatous infiltration. Second, plasma cell dyscrasia (e.g., multiple myeloma or plasmacytoma) was ruled out by the normal serum immunofixation electrophoresis test, normal κ/λ light chain ratio, and the absence of monoclonal spikes or lytic bone lesions upon imaging. Third, systemic autoimmune diseases such as systemic lupus erythematosus (SLE) or ANCA-associated vasculitis were considered unlikely, given the negative anti-dsDNA, negative ANCA, absence of skin rash or arthritis, and the predominant tubulointerstitial rather than glomerular pathology. Moreover, the low C3/C4 levels in this case were interpreted as part of the IgG4-RD-related complement consumption rather than evidence of SLE. Fourth, other causes of tubulointerstitial nephritis, including drug-induced, sarcoidosis, and infectious etiologies, were excluded based on a thorough consideration of the patient’s medication history (no nephrotoxic agents), normal serum angiotensin-converting enzyme levels, absence of granulomas on biopsy, and negative infectious workup. Finally, the combination of a markedly elevated serum IgG4 level, dense IgG4-positive plasma cell infiltration with an IgG4/IgG ratio >40 %, and characteristic storiform fibrosis on renal biopsy, along with the patient’s rapid clinical improvement following the administration of glucocorticoids, strongly supported IgG4-TIN as the primary diagnosis. Therefore, while the patient had concurrent IgA nephropathy and DN, it was considered that the patient’s acute renal deterioration was predominantly attributed to IgG4-TIN.

The therapeutic goal of IgG4-TIN is to reduce inflammation, induce remission, and preserve organ function. Glucocorticoids are the primary first-line treatment. In cases where the response to such treatment is inadequate, or where there is relapse or steroid intolerance, combination therapy with immunosuppressants or biologics, such as rituximab (an anti-CD20 monoclonal antibody), may be used. In the present case, the patient demonstrated a positive initial response to glucocorticoids, showing a decrease in creatinine levels from 817 μmol/L to 305 μmol/L. However, the patient’s serum IgG4 levels remained significantly elevated after steroid tapering to 7.5 mg/day, despite going from 13.89 g/L to 4.55 g/L. Moreover, the patient continued to experience fatigue, and his complement C3 levels remained persistently low. Given the low likelihood of maintaining long-term disease remission with low-dose steroid monotherapy and the potential for rituximab to reduce the risk of future relapse, the decision was made to incorporate rituximab as a consolidation therapy. The patient’s young age, multisystem involvement (lymphadenopathy, renal impairment), and the potential for steroid-related adverse effects with prolonged use were also considered in this decision-making process. Regarding rituximab, numerous studies have shown that it can effectively induce clinical remission and significantly lower serum IgG4 levels [16], 18], and it is currently recommended as a second-line therapy [19]. Standard treatment protocols with rituximab include an intravenous infusion of 375 mg/m^2^ body surface area weekly for 4 weeks, or 1,000 mg every 2 weeks with a 2-week gap between doses. Additional treatment courses can be repeated as needed to maintain disease control. However, the natural history and prognosis of IgG4-TIN are not fully understood, and there is a risk of relapse after stopping treatment; hypocomplementemia, for instance, is one identified risk factor for recurrence. Therefore, patients should undergo clinical evaluation every 3–6 months, with their serum IgG4 levels monitored to assess treatment effectiveness [2].

Recent literature increasingly supports the use of rituximab in IgG4-RKD, both as second-line therapy and as a steroid-sparing agent. For instance, a systematic review by Patel et al. [19]. that included IgG4-RD patients confirmed that rituximab could effectively induce clinical remission and significantly reduce serum IgG4 levels, while also benefiting from a favorable safety profile. Also, Peyronel et al. [18]. further emphasized that rituximab is particularly valuable in relapsed or refractory cases and in patients with contraindications to glucocorticoids. However, most published series to date have focused on pure IgG4-TIN, with limited data on patients with concurrent glomerular diseases. To date, only a few case reports have described IgG4-TIN overlapping with IgA nephropathy, and even fewer with additional DN. For instance, Chaba et al. [16].’s study involved a cohort of 83 IgG4-RKD patients, among whom a small subset had mesangial IgA deposition, but their treatment outcomes were not separately analyzed. In another report, Buglioni et al. [14]. described the clinicopathologic features of IgG4-RKD in a large cohort, yet the detailed therapeutic responses to rituximab in overlapping phenotypes were not provided. Our case contributes to this limited evidence base by documenting a case involving 32-month follow-up after rituximab consolidation (cumulative 1.8 g) in a patient with biopsy-proven IgG4-TIN, IgA nephropathy, and DN. Unlike previous reports where rituximab was often used for relapse or steroid resistance, our patient received rituximab as planned consolidation after his initial glucocorticoid response, with the aim to maintain remission and facilitate steroid tapering. The observed sustained reduction in serum IgG4 levels (from 13.89 g/L to 0.89 g/L) and stabilization of the patient’s renal function (creatinine ∼ 215 μmol/L) over two years suggest that this approach may be feasible even in complex overlapping renal pathology. Nevertheless, as a single case, it does not establish the superiority of this approach but rather highlights the need for future studies to specifically address the efficacy of rituximab in IgG4-RKD with concomitant glomerular diseases.

The presented case in this case report involved multisystem engagement, including lymphadenopathy, renal impairment (proteinuria, AKI), hematologic abnormalities (anemia, thrombocytopenia, eosinophilia, elevated IgG/IgE, decreased complement), hyper-IgG4 syndrome, and radiographic kidney abnormalities. Furthermore, renal biopsy showed lymphoplasmacytic infiltration, IgG4+ plasma cell proliferation, and striated fibrosis, confirming the diagnoses of IgG4-TIN, IgA nephropathy, and diabetic nephropathy. After glucocorticoid therapy and rituximab consolidation, the patient’s renal function improved gradually, with a significant reduction in serum IgG4 levels observed. At the last follow-up on March 28, 2026, the patient’s serum creatinine levels were relatively stable at around 215 μmol/L. Prior research has suggested that IgG4-related membranous glomerulonephritis may respond less favorably to treatment compared to IgG4-TIN [20]. Furthermore, if immunosuppressive therapy does not work, some patients might need dialysis or a kidney transplant. In the present case, the patient’s renal function has not yet fully recovered to date, necessitating the ongoing monitoring of their renal function, IgG4 levels, and complement markers. Long-term follow-up data from this case provide valuable insights for managing the disease. As a single case report, these findings cannot establish the comparative efficacy of our approach but support the clinical rationale for individualized therapy. Furthermore, the exact role of IgG4 in the disease’s development remains unclear and needs further research.

## Conclusions

This case underscores the significance of maintaining diagnostic vigilance for IgG4-RD in patients with unexplained multisystem involvement and acute kidney injury. The value of renal biopsy in confirming overlapping pathologies, including IgG4-TIN, IgA nephropathy, and DN, is underscored by this study. The clinical response observed with glucocorticoids followed by rituximab consolidation suggests that such a strategy may be considered in selected patients, although treatment decisions should be individualized. Furthermore, it is to be noted that the aim of this report was not to advocate for a universal standard; rather, this case report provides instructive experience for consideration in clinical practice.
